# Phenotypic Landscape of *Saccharomyces cerevisiae* during Wine Fermentation: Evidence for Origin-Dependent Metabolic Traits

**DOI:** 10.1371/journal.pone.0025147

**Published:** 2011-09-16

**Authors:** Carole Camarasa, Isabelle Sanchez, Pascale Brial, Frédéric Bigey, Sylvie Dequin

**Affiliations:** 1 INRA, UMR1083, Montpellier, France; 2 SupAgro, UMR1083, Montpellier, France; 3 Université Montpellier 1, UMR1083, Montpellier, France; University of Nebraska, United States of America

## Abstract

The species *Saccharomyces cerevisiae* includes natural strains, clinical isolates, and a large number of strains used in human activities. The aim of this work was to investigate how the adaptation to a broad range of ecological niches may have selectively shaped the yeast metabolic network to generate specific phenotypes. Using 72 *S. cerevisiae* strains collected from various sources, we provide, for the first time, a population-scale picture of the fermentative metabolic traits found in the *S. cerevisiae* species under wine making conditions. Considerable phenotypic variation was found suggesting that this yeast employs diverse metabolic strategies to face environmental constraints. Several groups of strains can be distinguished from the entire population on the basis of specific traits. Strains accustomed to growing in the presence of high sugar concentrations, such as wine yeasts and strains obtained from fruits, were able to achieve fermentation, whereas natural yeasts isolated from “poor-sugar” environments, such as oak trees or plants, were not. Commercial wine yeasts clearly appeared as a subset of vineyard isolates, and were mainly differentiated by their fermentative performances as well as their low acetate production. Overall, the emergence of the origin-dependent properties of the strains provides evidence for a phenotypic evolution driven by environmental constraints and/or human selection within *S. cerevisiae*.

## Introduction

Despite the extensive diversity of *S. cerevisiae*, most work on this model organism has been carried out using only a handful of domesticated laboratory strains. Since the discovery and identification of yeast as a fermentative microorganism in the 19^th^ century, a large number of *S. cerevisiae* strains have been isolated from diverse sources all over the world, corresponding to extremely different living environments. These include natural habitats of yeast in fruits, soil, cacti and the bark of oak trees, as well as the facultative infections of immunocompromised patients. However, *S. cerevisiae* is found most often in connection with human activities, which include baking, brewing, winemaking and fermented beverage production (sake, palm wine). Indeed, this yeast has been exploited by man for millennia for the fermentation and preservation of beverages and food [1], [2].

Recent advances in genomic tools allow the genetic diversity of *S. cerevisiae* to be assessed in unprecedented detail. The overall genetic variation between strains has been estimated to be between 0.1 and 0.5%, based on approaches using multilocus sequence typing, multilocus microsatellite analysis, genome sequencing and whole-genome tiling arrays [3], [4], [5], [6], [7]. Specific and large-scale genome sequencing projects have resulted in a massive amount of genomic data for *S. cerevisiae* [8], [9], [10], [11]. Phylogenetic analysis of strains from a broad-range of ecological niches, revealed that *S. cerevisiae* originated in a wild habitat, probably the bark of oak trees, and that a subset of strains specialized for fermentation were emerged from subsequent selection and cultivation [4]. In addition, domestication events, rather than geography, substantially impacted the genetic structure of the *S. cerevisiae* population [7], [8], [12], [13], [14], [15], [16]. These domestication events were followed by human-associated dissemination of these yeasts throughout the world.

To date, the phenotypic variation of yeast populations originating from diverse environments has been only partially characterized. Several studies have focused on identifying the genetic bases for specific physiological traits, such as high-temperature growth [17], [18], ethanol resistance [19], sporulation efficiency [20] [21], drug responses [22], [23] and morphology [24]. These studies generally concerned growth determinations for a limited number of laboratory or vineyard strains or clinical isolates. Recently, extensive phenotypic variation in the mitotic proliferation ability of strains, was reported following high-throughput stress resistance analysis or adaptation to diverse environments (carbon and nitrogen sources, presence of toxins, nutrient limitations) for collections of *S. cerevisiae* strains [8], [25] [26], [27].

The variability between strains for phenotypes other than growth, particularly for metabolic traits such as glycolytic flux, metabolite yields, or the ability to use various substrates, has been poorly investigated despite their considerable industrial interest. In connection with this, eight strains with diverse genetic backgrounds (laboratory strains, vineyard and clinical isolates) were reported to be highly variable for a simple phenotypic trait, namely the utilization of di/tripeptides as nitrogen source [28]. Similarly, a population of 19 strains assembled from five different habitats (industry, forest, laboratory, clinic, fruit) exhibited an important variability both in life-history traits of yeast growth and in metabolic traits (glycolytic rate and ethanol production) [29]. More recently, the diversity between 9 food-processing strains (brewery, enology, distillery) has been analyzed regarding their growth and metabolic behaviors in three industrial processes [30].

In view of the limited information available, the natural genetic resources of *S. cerevisiae* and the phenotypic variations between strains, and particularly those related to metabolism, bear further systematic exploration. The first aim of this study was to assess the extensive diversity of *S. cerevisiae* yeast strains by investigating a large panel of yeasts with respect to their specific phenotypic traits. A special attention was paid to phenotypes that have been directly the target of human selection for industrial purposes such as fermentation performance and kinetics, production of acetate, glycerol and aromatic compounds. Strains from diverse sources and environments (clinical, industrial, laboratory and wild isolates) were grown under the conditions of wine fermentation. These conditions are characterized by high sugar and ethanol concentrations, high acidity, low nitrogen availability, and anaerobiosis. Since wine fermentation can be regarded as an extreme environment expected to highlight variations between strains, it constitutes a model system for studying yeast phenotypic diversity.

The wide variety of the environments from which these strains were collected represent an array of conditions and stressors which likely contributed to the emergence and divergence of different phenotypes. These arose as the organisms developed distinct strategies to face the selective pressures of their living environments. In addition, fermentation yeasts typically have been specialized for a particular industrial process (e.g., baking, brewing, winemaking, etc.) through human manipulation. Consequently, both environmental and human selective pressures may have resulted in specific properties being shared by strains which live in similar habitats. We also analyzed the resulting phenotypic dataset to determine whether some traits were specific to a particular ecological niche. This allowed us to investigate the relationships between yeasts and their environments and to assess whether the evolution of certain phenotypes was driven by environmental and/or human factors.

## Results

### Strain phenotypes under extreme fermentation condition

To investigate the phenotypic diversity among the *S. cerevisiae* strains, we characterized the fermentation performances of 72 strains obtained from widely different environments and sources (Table 1). The strains included the reference strain, S288C, and other lab strains (8), natural strains (19), clinical isolates (13), yeasts used in fermentative processes (10), yeasts found in vineyards (8) or in commercial winemaking (9), and several baker's yeast (5) strains. Anaerobic fermentations were carried out in synthetic MS medium containing a high glucose concentration and limited amounts of nitrogen and lipids. Although anaerobiosis was not imposed, it occurred rapidly and spontaneously due to the design of fermentors and the large amount of CO_2_ produced. Under these conditions, yeast proliferation was rapidly limited by the low nitrogen concentration in the medium, so that most of the sugar was consumed by resting cells during the stationary phase. Throughout the fermentation process, the CO_2_ production rate increased rapidly as the number of cells increased, then progressively decreased during the stationary phase. To simplify the analysis of the complex fermentation rate profiles, five variables were extracted from each fermentation curve. These included the total amount of released CO_2_ (CO_2F_), and four kinetics variables: the maximal fermentation rate (V_max_), the time at which 50% and 75% of sugars were consumed, as estimated by 55 and 80 g/L of CO_2_ produced (T_50_ and T_75_, respectively) and the fermentation rate at T_50_ (V_50_). In addition, the phenotypic description of each strain consisted of two growth features (dry weight, population size) and 11 metabolic variables (glycerol, acetate, succinate and eight volatile organoleptic compounds), measured at T_75_. This resulted in a data set of 18 variables for each of the 72 strains.

**Table 1:** Collection of *S. cerevisiae* strains from diverse environments and geographical locations.

Environment	Strain	Geographical origin	Comments	Collection
Baker				
	CLIB 324	Saigon, Vietnam	Baker strain	Washington
	CLIB 215	New Zealand	Baker strain	Washington
	YS2	Australia	Baker strain	Sanger
	YS4	Netherlands	Baker strain	Sanger
	YS9	Singapore	Baker strain, Le Saffre	Sanger
Clinical				
	YJM280	USA	Peritoneal fluid	Washington
	YJM320	USA	Blood	Washington
	YJM326	USA	Patient	Washington
	YJM421	USA	Ascites fluid	Washington
	YJM653	USA	Broncho-alveolar lavage	Washington
	273614N	Newcastle UK	Fecal isolate	Sanger
	322134S	Newcastle UK	Throat-sputum isolate	Sanger
	378604X	Newcastle UK	Sputum isolate	Sanger
	YJM428	USA	Paracentesis fluid	Washington
	YJM451	Europe	Patient	Washington
	YJM975	Bergamo, Italy	Vaginal isolate	Sanger
	YJM978	Bergamo, Italy	Vaginal isolate	Sanger
	YJM981	Bergamo, Italy	Vaginal isolate	Sanger
Fermentation processes				
Beer				
	CLIB 382		Beer	Washington
	NCYC361	Ireland	Beer spoilage strain from wort	Sanger
Palm wine				
	Y12	Ivory Coast, Africa	Palm wine,	Washington
	DBVPG1853	Ethiopia	White Tecc	Sanger
	DBVPG6044	West Africa	Bili wine	Sanger
	NCYC110	Nigeria, West Africa	Ginger beer from Z. officinale	Sanger
	PW5	Africa	Raphia palm wine	Washington
Sake				
	K11	Japan	Shochu sake strain Awamori-1	Sanger
	UC5	Japan	Sene sake	Washington
	Y9	Japan	Ragi (similar to sake wine)	Sanger
Laboratory				
	FL 100	France	Crossing from D2339-17 and S1786	Washington
	CEN.PK	Germany		INSAT^a^
	ENY.WA-1A p			
	S288c	California, USA	Rotting fig	Sanger
	SK1	USA	Soil	Sanger
	W303	USA		Sanger
	W303 p	USA		
	Y55	France	Wine	Sanger
Natural				
Bertam palm				
	UWOPS03-461.4	Malaysia	Nectar, Bertam palm	Sanger
	UWOPS05-217.3	Malaysia	Nectar, Bertam palm	Sanger
	UWOPS05-227.2	Malaysia	Trigona, Bertam palm	Sanger
Cactus				
	UWOPS83-787.3	Bahamas	Fruit, *Opuntia stricta*	Sanger
	UWOPS87-2421	Hawaii	Cladode, *Opuntia megacantha*	Sanger
Fruit				
	Y10	Phillipines	Coconut	Washington
	CBS 7960	Sao Paulo, Brazil	Produces ethanol from cane-sugar syrup.	Washington
	DBVPG6040	Netherlands	Fermenting fruit juice	Sanger
	DBVPG6765	Indonesia	Lici fruit	Sanger
Oak				
	NC-02	North Carolina, USA	Oak tree exudates	Washington
	T7	Missouri, USA	Oak tree exudates	Washington
	YPS1009	New Jersey, USA	Oak tree exudates	Washington
	YPS128	Pennsylvania, USA	Oak tree exudates	Sanger
	YPS163	Pennsylvania, USA	Oak tree exudates	Washington
	YPS606	Pennsylvania, USA	Oak tree exudates	Sanger
Soil				
	DBVPG1373	Netherlands	Soil	Sanger
	DBVPG1788	Finland	Soil	Sanger
	I14	Italy	Soil sample	Washington
	IL-01	Illinois, USA	Soil sample	Washington
Vineyard				
	YJM269	Portugal	Blauer Portugieser grapes	Washington
	BC187	Napa Valley, USA	Barrel fermentation, haploïd derivative UCD2120	Sanger
	DBVPG1106	Australia	Grapes	Sanger
	L-1374	Chile	Wine	Sanger
	L-1528	Chile	Wine	Sanger
	M22	Italy	Vineyard isolate	Washington
	YIIc17_E5	France	Sauternes wine	Sanger
	RM 11	California, USA	Haploïd derivative Bb32	Washington
Wine commercial				
	59-A	France	Meiotic spore of strain EC1118	
	V5p	France	Meiotic spore of strain CIVC8130	
	T73	Spain	Red wine ( Monastrel)	Lalvin
	71B	Germany	Vineyard	Lalvin
	EC1118	France	Champagne fermentation	Lalvin
	L2226	France	Vineyard (Côte du Rhone)	Enoferm
	WE372	South Africa	Wine (Cape Town)	Anchor
	K1M	France	Grapes	Lalvin
	VL1	France		Laffort

a Professeur Jean-Marie François, Laboratoire Ingenierie des Systèmes Biologiques et des Procédés, Institut National des Sciences Appliquées, Toulouse.

According to data on the habitats of yeasts, mainly from Sanger Institute and Washington University databases, strains were first classified into 7 major groups: Baker, Clinical, Fermentation processes, Laboratory, Vineyard, Natural and Wine commercial. The *Fermentation processes* and *Natural* clades were further differentiated into three and five subgroups, respectively.

We tested the reproducibility of our phenotypic analysis by fermenting several strains at least in duplicate. For each of these strains, the fermentation profiles were almost identical (Figure S1) and could be considered as a fingerprint of the strain's performance under standardized culture conditions. Moreover, we detected no substantial variation between the independent determinations of the fermentation kinetics, growth and metabolic variables and the intra-class correlation coefficients ranged between 86% and 99%, with a mean value of 95%. (Table S1). This reproducibility analysis indicated the feasibility of our approach for assessing phenotypic diversity in *S. cerevisiae*.

### Phenotypic variations among *S. cerevisiae* strains

**Figure 1:**
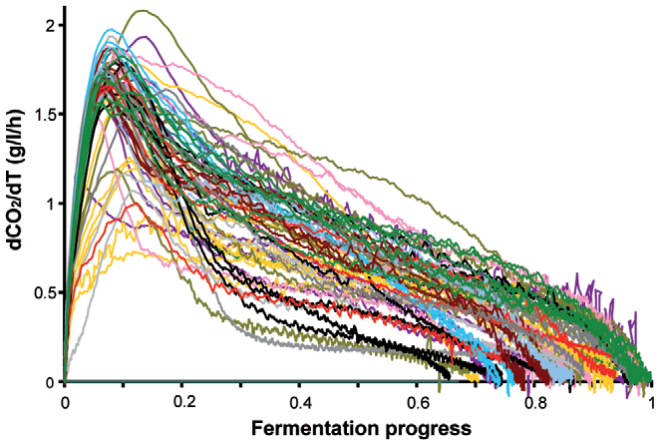
Comparison of the fermentation profiles for 72 *S. cerevisiae* strains fromdiverse geographical locales and environments. Fermentations were carried out in synthetic medium containing 240 g/L glucose, 200 mg/L nitrogen, pH 3.5. The fermentation profiles are presented as the CO_2_ production rate vs. the fermentation progress, which corresponded to the ratio of consumed glucose to initial glucose. The lines are colored according to the origin of the strains: vineyard (purple), soil (grey), sake (light grey), palm wine (pink), oak (brown), laboratory (yellow), fruit (red), wine commercial (dark green), Bertam-palm (blue), baker (black), beer (dark grey), cactus (light blue).

The fermentation profiles of the 72 strains varied substantially from each other, reflecting their diverse fermentative performances (Figure 1). Many of the 72 strains were able to complete the fermentation of 240 g/L glucose. However, 45% of them exhibited a stuck profile and stopped fermenting before glucose was exhausted (i.e., the residual glucose concentration was above 10 g/L and CO_2_ production was below 105 g/L). Great variations in kinetics variables were observed between the strains. For example, V_max_ was between 0.4 and 2.1 g/L/h and T_75_ was between 64 and 444 h. Due to the broad diversity in the origins of the strains, the value ranges for the measured variables, especially for V_max_, were considerably greater than what was previously reported for commercial wine yeasts [31], [32] or strains from industry (distillery, wine, bakery) [30]. Nevertheless, for most strains, V_max_, T_50_, and to a lesser extent, T_75_ were very similar and only a few individuals exhibited extreme behaviors. The values for the V_50_ variable, which described the activity of the yeast during the latter stages of fermentation, were more dispersed than other variables, and were predominantly between 0.2 and 1.2 g/L/h. This observation suggested that considerable diversity exists in the abilities of yeast to face the multiple stresses conditions present at the end of fermentation (e.g., ethanol toxicity, nitrogen and micro-nutrient starvation).

**Figure 2:**
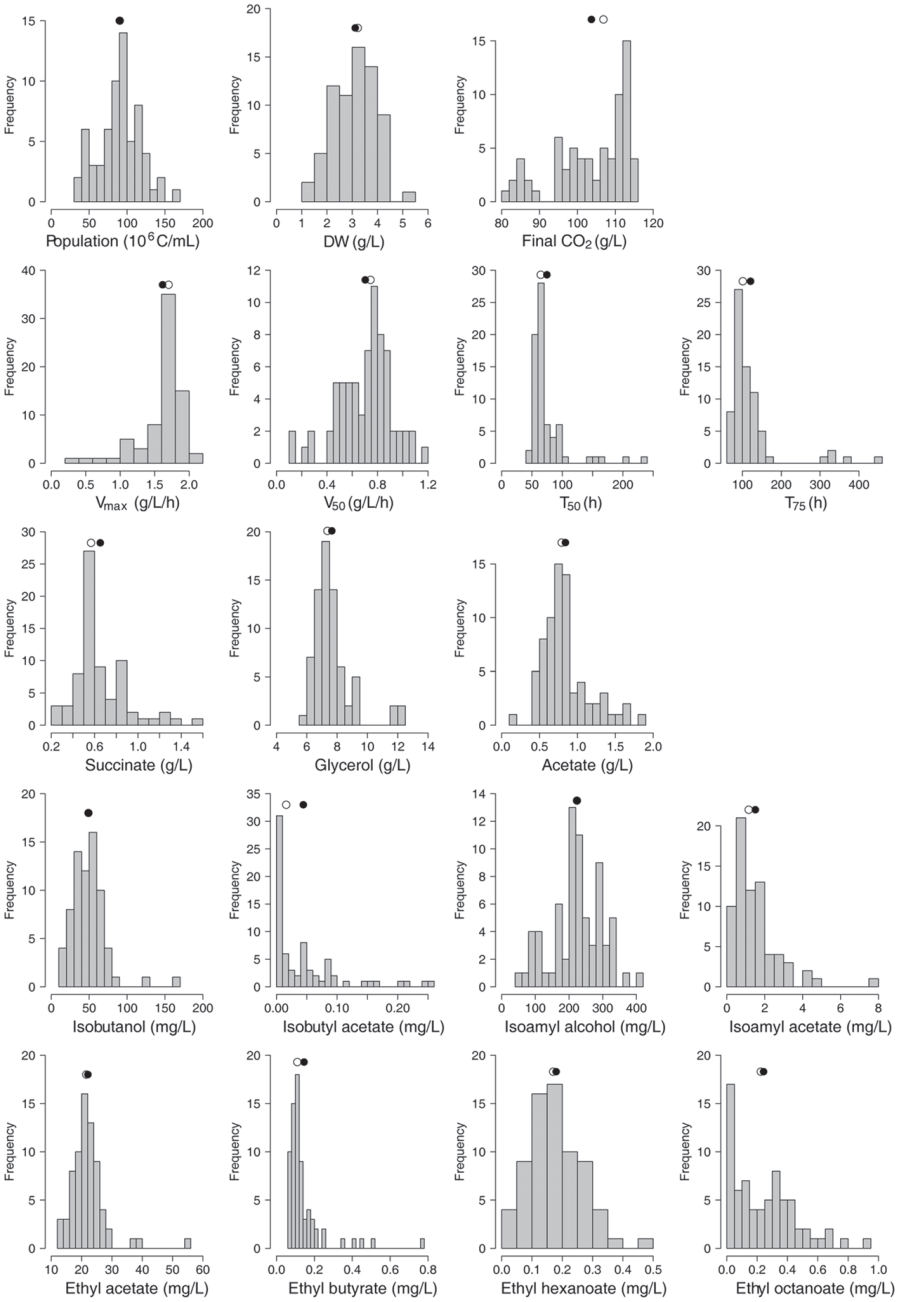
Distribution frequency of the phenotypic variables in the total population (72 strains). Closed circle: mean value; open circle: median value. Kinetics variables (CO_2F_, V_max_, V_50_, T_50_ and T_75_) were determined from the fermentation curves. Growth (cell number and dry weight) and metabolic variables (glycerol, acetate, succinate, two higher alcohols, two acetate esters and four ethyl esters) were measured when 75% of the sugars were consumed (180 g/L).

Considerable differences between strains, which were between 2- and 15-fold, were also found in the formation of biomass (dry weight) and in the synthesis of fermentation by-products (except ethanol). A large part of the variables was symmetrically distributed about the mean (Figure 2), although a few outliers existed for strains displaying extreme production levels (such as the synthesis of 12 g/L glycerol by CBS7960 and NCYC361). Positive skewed or reverse J-shaped distributions were observed for the production of acetate esters and some ethyl esters derivatives, reflecting the null or limited production of these compounds by the majority of the studied strains. Finally, in contrast to the great differences found in the formation of other metabolites, the conversion of glucose to ethanol remained almost constant among the whole population as usually viewed, with a mean value of 0.46±0.01 g ethanol/g glucose.

### Relationships between the phenotypic traits

**Figure 3:**
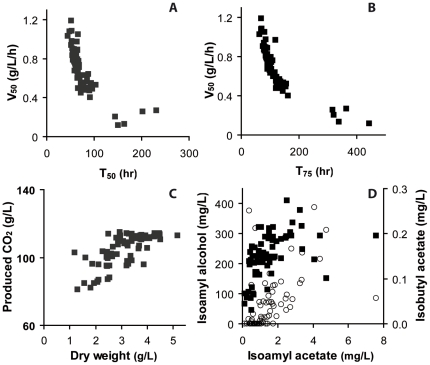
Relationships between the phenotypic variables within the total population of strains. Correlations were found between: the fermentative activity at mid-fermentation V_50_ and the time necessary to consume 50% or 75% of sugars, T_50_ (A) and T_75_ (B), respectively; the final CO_2_ release and the dry weight (C); and the production of isoamyl acetate and the productions of isoamyl alcohol (▪) and isobutyl acetate (○) (D).

Considering the size of the population, we used the Pearson's Product Moment method to perform a correlation analysis between all the phenotypic traits (Tables S2, S3). Most of the variables varied independently within the population. However, as expected, T_75_ and T_50_ were strongly correlated (r = 0.92, p<0.001) with each other and both of them were correlated negatively with V_50_ (r = −0.81, p<0.001 and r = −0.78, p<0.001, respectively, Figures 3A & B). The maximal fermentation rate V_max_ and the kinetics variables characterizing the last steps of the fermentation process (V_50_ and T_75_) were moderately but significantly correlated (r = 0.48, p<0.001 and r = −0.52, p<0.001, respectively). This strong correlation significance indicated that V_max_, which measures activity at the beginning of the process (growth phase), impacted at least partially the behavior of the strain during the stationary phase (end of fermentation). However, the fairly weak correlation coefficients, close to 0.5, suggested a partial decoupling between the first and the last parts of fermentation, likely due to the contribution of other parameters in the control of the stationary phase, as the tolerance of the strains to inhibitory compounds (ethanol). A correlation (r = 0.71, p<0.001) was found between the biomass production and the final amount of CO_2_ released, indicating that most poorly growing strains exhibited stuck profiles (Figure 3C). This is consistent with previous reports citing the inability of commercial yeasts to complete wine fermentation when nutrient limitations affect their growth [33], [34], [35], [36]. Regarding the metabolic variables, substantial correlations were found between the productions of isoamyl acetate and either its isoamyl alcohol precursor (r = 0.49, p<0.001) or isobutyl acetate (r = 0.51, p<0.001), which is the other main acetate ester produced by yeast during alcoholic fermentation (Figure 3D).

### Metabolic traits discriminate strains from different origins

To identify potential relationships between strain origin and fermentation phenotype, the data set was first analyzed regarding seven ecological niches: baker, clinical, fermentation processes, laboratory, vineyard, natural and wine commercial (Figure S2, Table S4). To obtain a general overview of the data, a principal component analysis [PCA] and a linear discriminant analysis [LDA] (with origin as factor) were first performed using all the traits and all the strains. Only 38% of the variation was explained by the two first components of the global PCA and the LDA analysis did not allow to discriminate the origin of strains, due to the complexity of the dataset (Figure S3). Consequently, an exploratory study was performed in order to select the variables that exhibited a significant global effect among the seven groups of strains (Table 2, Table S5). Univariate analyses of variance (ANOVA) without multiplicity adjustment identified several variables relevant to discriminating the strains on the basis of their origin (p-value<0.05): dry weight, population size, CO_2F_, T_75_, T_50_, glycerol, acetate and ethyl butyrate. For the other descriptive variables, variations were mainly attributed to the large intra-group variability. The analysis of the most selective variables allowed us to identify specific traits common to all the strains from the same ecological niche, for three groups: wine commercial, baker and laboratory. Indeed, a noteworthy characteristic of laboratory strains was their high level of ethyl butyrate synthesis compared to strains from other habitats. In addition, we found that these yeasts produced little biomass, fermented sugars slowly, produced high amounts of acetate and low amounts of isoamyl acetate. Conversely, commercial wine strains were able to completely and rapidly ferment the available sugars, while producing high biomass and little acetate. Finally, we found that bakery yeasts were characterized by low acetate, succinate and glycerol productions but, contrary to wine commercial strains, exhibited poor growth and fermentative performances.

**Table 2:** Univariate analysis of the variance for the phenotypic variables.

Phenotypic variable	p-value7 groups	p-value13 groups
Cell number	0.035	0.15
Dry weight	0.004	0.002
V_max_	0.06	0.004
V_50_	0.09	0.005
T_75_	0.02	0.0006
T_50_	0.01	<0.0001
CO_2F_	0.02	0.0005
Glycerol	0.03	0.001
Acetate	0.001	0.04
Succinate	0.65	0.46
Isobutanol	0.12	0.22
Isobutyl acetate	0.34	0.33
Isoamyl alcohol	0.34	0.46
Isoamyl acetate	0.06	0.16
Ethyl acetate	0.08	0.08
Ethyl butyrate	<0.0001	0.0004
Ethyl hexanoate	0.090	0.007
Ethyl octanoate	0.71	0.14

No adjustment of multiplicity.

The phenotypic traits were measured for 72 strains classified into 7 or 13 groups of origin.

For the other origins (vineyard, clinical, nature, fermentation processes), we found a large intra-group variability for all the phenotypic traits. This may be explained by the intrinsic diversity within each class. The clinical strains were isolated from human infections. Since these yeasts are generally considered to originate from other environments [7], [37], substantial phenotypic variability can be expected in this group. The nature, vineyard and fermentation processes groups consisted of strains from habitats with a strong heterogeneity regarding the living conditions. The nature group also consisted of strains from different environments, including sugar-rich and sugar-poor ones, which may have likely affected their cell physiology. Consequently, we redefined these categories (Table 1) by separating the nature isolates into fruit, cactus, Bertram palm, oak and soil subgroups and the fermentation processes group into beer, sake, and palm wine. In this way, a total of 13 categories were established and analyzed as described above. This reclassification substantially decreased intra-group variability for most of the phenotypic traits (Figure S2). The two most significant variables for discriminating the strains among the 7 groups, namely ethyl butyrate and acetate, as well as the kinetic variables (T_75_, T_50_, V_50_, V_max_ and CO_2F_), the dry weight and the production of ethyl hexanoate were identified as contributed substantially to the variance between the 13 habitats (Table 2). Palm wine strains consumed sugar at high rates throughout the fermentation course, resulting in short fermentation times. These strains were specifically differentiated by their low succinate production and, to a lesser extent, by their high acetate and isoamyl acetate production. Yeasts used for sake and beer fermentation exhibited low fermentation rates (V_50_) and long fermentation times. Beer strains were distinguished from sake strains and all other strains, by their low biomass production and by their low levels of aromatic compound synthesis, particularly of the ethyl ester derivatives (ethyl hexanoate). Regarding the nature group, all the strains isolated from soil and fruits were able to complete the fermentation of sugar, unlike the strains derived from cactus, oak and palm habitats. These strains were further discriminated by biomass production, which was high for cactus and fruit strains and low for oak and palm strains. Furthermore, the profile of ethyl ester synthesis during fermentation varied greatly among these groups. Oak and Bertam-palm strains produced low levels of ethyl acetate compared to the other strains, whereas cactus and Bertam-palm strains produced high levels of ethyl octanoate. Ethyl hexanoate production was high for the soil and oak strains and low for the Bertam-palm strains.

### Phenotypic differentiation of commercial wine from vineyard yeasts

**Figure 4:**
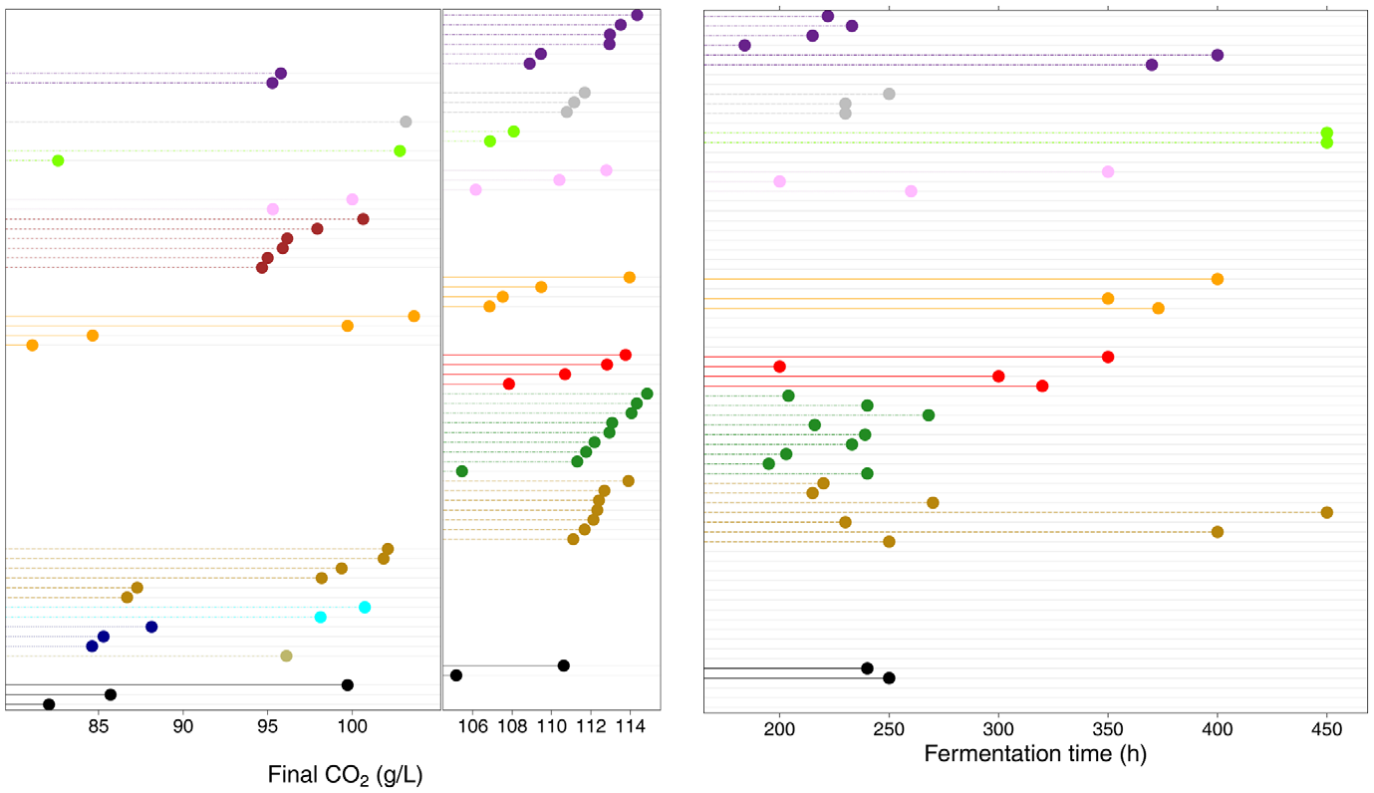
Abilities of the 72 strains from different environments to efficiently ferment a high concentration of sugar. Strains were considered able to achieve fermentation of 240 g/L of sugar when their production of CO_2_ at the end of fermentation (A) was higher than 105 g/L. These strains were further discriminated by the fermentation time (B). Vineyard: purple symbols; soil: grey symbols; sake: green symbols; palm: pink symbols, oak: brown symbols; laboratory: yellow symbols; fruit: red symbols; wine commercial: dark green symbols; clinical: tan symbols, cactus: blue symbols; Bertam –palm wine: dark blue symbols; beer: beige symbols ; baker: black symbols.

We compared the specific phenotypic properties of the wine commercial strains to those of yeasts from other environments, particularly the vineyard strains. The wine commercial strains, as well as strains originating from fruits and the majority of those from the soil (3/4) and vineyard (6/8) groups, were able to completely ferment 240 g/L glucose (Figure 4A). Whereas all the wine commercial strains had short fermentation times (<270 h), some individuals in the three other groups of strains having good fermentative capacities, including the vineyard set, displayed prolonged fermentation profiles (Figure 4B). More generally, the variability of wine commercial strains was lower than that of the vineyard group (Figure S2, Table S4), and substantially lower standard deviation values than those of the vineyard group were observed (Table 3). In addition, the wine commercial strains exhibited extreme values for some specific phenotypic traits compared to those of vineyard strains. These included production of acetate (0.6±0.1 g/L versus 0.9±0.4 g/L, respectively), of isoamyl alcohol (and its acetate ester derivative) (242±64 g/L versus 194±87 mg/L, respectively) and V_50_ (0.8 g/L·h±0.1 versus 0.7 g/L·h±0.2, respectively). All together, these results showed that wine commercial strains constituted a minimally diverse subset of yeasts from vineyard, in agreement with the selection of these strains for their technological traits by man.

**Table 3:** Comparison of the phenotypic variables between strains isolated from the wine commercial and vineyard strain groups.

Phenotypic variable	Wine commercial		Vineyard	
	Mean	S.D.	Mean	S.D.
Cell number, 10^6^ c/mL	119	27	92	22
Dry weight, g/L	3.6	0.5	3.8	0.9
V_max_, g/L/h	1.7	0.2	1.6	0.3
CO_2_produced g/L	112	3	108	8
T_75_ hr	88	10	109	27
T_50_ hr	61	5	67	16
V_50_ g/L·h	0.8	0.1	0.7	0.2
Succinate g/L	0.62	0.14	0.63	0.29
Glycerol g/L	7.0	0.4	7.1	0.7
Acetate g/L	0.6	0.1	0.9	0.4
Isobutanol g/L	52	7.8	70	48
Isobutyl acetate g/L	0.04	0.04	0.08	0.06
Isoamyl alcohol g/L	242	64	194	87
Isoamyl acetate g/L	1.9	1.2	1.3	0.7
Ethyl acetate g/L	24	3	23	3
Ethyl butyrate g/L	0.18	0.13	0.13	0.04
Ethyl hexanoate g/L	0.2	0.1	0.2	0.1
Ethyl octanoate g/L	0.2	0.2	0.3	0.3

### Effects of strains origin on phenotypic profiles

**Figure 5:**
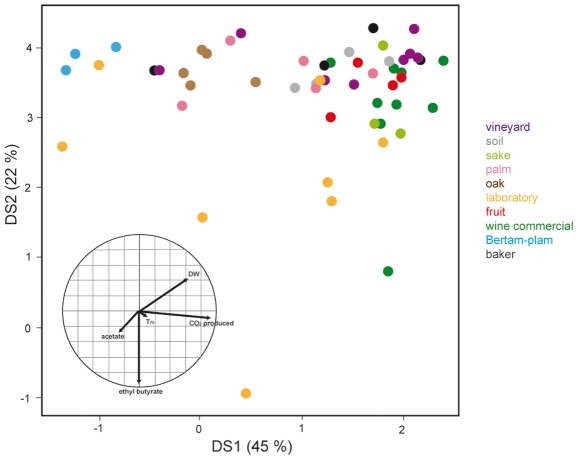
Linear discriminant analysis (LDA) of the population based on five discriminating phenotypic traits. A linear discriminant analysis was applied to the most discriminating variables: dry weight, T_75_, CO_2F_, acetate and ethyl butyrate measured for 53 strains representing 10 different groups. Clinical isolates were not included, due to the large phenotypic variability observed among the strains in this group. *Beer* and *cactus* groups, with only two strains each, were removed for this analysis. Groups of origin include vineyard (purple), soil (grey), sake (green), palm wine (pink), oak (brown), laboratory (yellow), fruit (red), wine commercial (dark green), Bertam-palm (dark blue), baker (black).

Finally, we carried out a comprehensive assessment of the relationships between their habitats (qualitative variable) and their quantitative phenotypic traits. A population of 57 strains from 10 different groups was initially considered for this analysis: laboratory, baker, wine commercial, sake, palm wine, vineyard, oak, soil, fruit, and Bertam palm. Clinical strains were excluded from the analysis because of their high intragroup variability, as well as *beer* and *cactus* groups, with only two individuals each. A linear discriminant analysis (LDA), was applied to the most discriminant phenotypic traits: dry weight, T_75_, CO_2F_, acetate, and ethyl butyrate. This analysis explained 45% and 22% of the intergroup variance in the first two discriminant axes, respectively (Figure 5, Figure S4).

The LDA first showed that the laboratory strains clearly separated from other individuals according to their high production of ethyl butyrate and, to a lesser extent, that of acetate. Moreover, the yeasts from rich-sugar environment, composed of the wine commercial (8/9), fruit (3/4) and vineyard (5/8) strains, were close to each other in the LDA representation on the basis of their high levels of CO_2_ production, which reflected their good fermentative capacities, high dry weights, and low levels of acetate production. Surprisingly, these strains displayed phenotypic similarities and clustered together with some sake, baker and soil yeasts. The univariate analysis showed that some of these groups of strains were discriminated on the basis of other specific variables (Figure S2), which were not included in the global LDA analysis.

Finally, two groups consisting of oak isolates on one hand and of strains from Bertam palm on the other hand, emerged due to their low dry weight and defective fermentation abilities. Together, these observations show that these few phenotypic traits, measured under these extreme fermentation conditions, allowed some strains to be discriminated based on their origin. However, this did not include yeasts from the soil and palm wine groups, which were not clearly distinguishable.

## Discussion

### Wine fermentation phenotypes reflect a wide diversity in yeast response to a stressful environment

The large data set of 18 kinetic, metabolic and growth characters determined during glucose fermentation for 72 *S. cerevisiae* strains (1296 distinct measurements) provides a detailed picture of the extent of metabolic diversity within this species, which has been poorly explored until now. Wine fermentation conditions represent a combination of various stresses (osmotic, ethanol, acidic, nutrient limitation) that accentuate the metabolic differences between strains. All variables except ethanol production varied independently within the population for most strains, and to a large extent (between 2- and 15-fold, depending on the variable). This substantial variability highlighted the disparate strategies used by the strains to cope with the numerous environmental stresses found in alcoholic fermentation and their different levels of ability to adapt to this extreme environment. Moreover, there was little or no correlation between the vast majority of variables, including metabolic ones, either within the entire population as a whole or within groups of strains separated according to their ability to complete fermentation or to their environmental origin. This suggests great inter-strain diversity in the metabolic strategies they used to deal with these unfavorable conditions, which derives from a substantial flexibility of the *S. cerevisiae* metabolic network.

A key factor that differentiated the strains was their ability to complete fermentation: 45% of the strains were unable to entirely consume the available sugars (240 g/L) and exhibited stuck fermentation profiles. Two main reasons for these problematic fermentations were identified in the case of commercial wine yeasts growing on grape juices. First, a nutrient limitation (e.g., nitrogen or lipids) may result in a low fermentation rate as a consequence of inefficient growth [33], [38], [39]. Second, the toxicity of fermentation by-products (e.g., ethanol and fatty acids) during the latter stages of fermentation may inhibit sugar transport and alter cellular membrane integrity, leading to reduced metabolic activity and viability [40], [41], [42], [43]. Consistent with the first hypothesis, we found that fermentation efficiency was related to biomass production. Furthermore, for all strains unable to complete the fermentation, the fermentation rate became asymptotic toward the end of the culture, which has been reportedly due to loss of viability [34]. This supports the main role of biomass production as the factor governing the fermentation course under stressful conditions. However, contrary to previous observations from wine commercial strains [34], [38] or from industrial environments [30], we found no correlation between V_max_ and biomass or population size. This is likely due to the use of highly diverse *S. cerevisiae* strains from a broader range of environments.

### Adaptation to environment results in emergence of specific metabolic traits

Shared phenotypes among strains from similar environments has been reported for yeasts collected from oaks, which exhibit freeze-thaw resistance crucial for survival in wintry environments, and for vineyard isolates, which have low sensitivities to copper sulfate, an anti-microbial agent widely used in European fields [5], [25], [44]. Similarly, the profile of resistance of sake-producing yeasts to various stresses was consistent with their specialized metabolism for growing under the defined conditions of sake fermentation [25]. Our study revealed additional specific traits that characterize strains originating from the same ecological niche. Examples include the low level production of fermentation by-products (e.g., glycerol, acetate and succinate) by baker's yeasts or the very high production levels of ethyl butyrate and limited biomass formation exhibited by laboratory strains. Surprisingly, in addition to wine commercial strains, most of the strains in the vineyard, fruit and soil groups also displayed good fermentative properties, whereas strains from oak, plant and brewery environments exhibited most of the stuck fermentation profiles.

Recently, an exhaustive mapping of the mitotic proliferation traits of *S. cerevisiae* growing under a wide-range of environments, reported a strong effect of population genetic history on trait variations within this species, suggesting that the relationships between ecological niche and phenotypes may be fortuitous or due to a common influence from a shared genetic lineage [27]. Our analysis, based on a population-scale phenotyping of *S. cerevisiae* restricted to only wine fermentation conditions but measuring a large number of growth, kinetics and metabolic characters allows to reveal links between source environments and specific phenotypes of some groups of strains. These relationships between the origin and the properties of some strain groups likely reflect phenotypes that evolved in response to environmental constraints. The stresses and conditions of particular habitats may have shaped the metabolism and physiology of these strains, resulting in adaptations and the emergence of environment-specific traits. Two different *S. cerevisiae* life-history strategies (grasshopper ant) have been previously defined on the basis of specific growth characteristics (rate, cell size, final population size) of strains related to the resources available in their original environment [29], [45].Similarly, the higher fermentative capacities of strains in the fruit, vineyard and wine commercial groups, may have arisen through selection in response to the prevalence of high sugar concentrations in these environments. Adaptations to osmotic stress and to the toxicity of fermentations products, such as the ethanol and fatty acids generated in the presence of abundant sugar, allow strains found in these environments to have selective advantages which contribute to their prevalence and to their capacity to efficiently ferment large amounts of sugar. Conversely, we found that yeasts isolated from “poor-sugar” environments (e.g. oaks and other plants) do not exhibit these efficient fermentation features. In the same way, laboratory strains, which have been propaged for many generations on rich media, optimal for growth [46], most likely lost their capacity to thrive in harsh environmental conditions. This might explain why they grew poorly and exhibited stuck profiles during alcoholic fermentation.

Human selection is another factor that has contributed to the environment-specific properties of strains used in industrial processes [13]. The physiologic and metabolic features common to all the wine commercial strains include low acetate production, substantial biomass production, aroma production (in the form of isoamyl alcohol and ester-acetate) and the fast and efficient fermentation of high sugar concentrations. These traits differentiate the wine commercial strains from strains found in other environments, including the vineyard strains. These phenotypes are the consequences of human selection since wine commercial strains have been intentionally picked out from vineyard environments and exploited for winemaking due to their advantageous kinetic and metabolic characteristics. Similarly, the low production of acetate, glycerol and succinate by-products exhibited by baker strains likely reflects the human selection of strains for their high CO_2_ production rates, which are needed for bread-making [10], [47] and are detrimental to by-product formation. The clade of laboratory strains, from which most *S. cerevisiae* knowledge has been acquired, was significantly differentiated from the other strain groups for many phenotypic traits, including low biomass formation, poor fermentation performances and synthesis of specific metabolites. This may be explained by the fact that most of the commonly used laboratory strains were derived from the S288C genetic background [7], [48], [49]. However, our analysis included two strains with S288C independent genetic background, SK1 and Y55. The divergence of all the laboratory strains with the rest of *S. cerevisiae* population may also reflect their long-term domestication under optimal growth conditions which likely repressed some protective and adaptive mechanisms essential for survival in natural environments [46].

In contrast to the other groups of strains, the phenotypes of the clinical strains were broadly distributed compared to those of the entire population. This may be explained by the opportunistic colonization of human tissues by *S. cerevisiae* strains which normally inhabit different environments, and thus differed considerably in their physiologic and metabolic traits. Consistent with our phenotypic observations, the clinical isolates were also highly diverse genetically and did not form a coherent group [7], [25], [37].

Overall, the low variability of laboratory strains compared to the total population highlights the need to continue to study the genomic and phenotypic diversity of *S. cerevisiae*. This research will provide new insights on the relationships and interactions between *S. cerevisiae* and its highly varied environments, and on the molecular mechanisms involved as yeasts adapt to their habitats. Ultimately, this will allow better use of this species ample natural genetic resources.

Genomic analyses of panels of *S. cerevisiae* strains identified distinct subgroups based on the identification of SNPs, which demonstrated that the *S. cerevisiae* population structure at least partly reflected the numerous different environments from which yeasts were isolated [7], [8]. Interestingly, it was recently reported that the genetic subgroup Wine/European could be differentiated from other lineages, namely Malaysian, West African and North American, based on the phenotypes of strains grown in different environments and in the presence of different drugs [8] [27], indicating that trait variations in yeasts reflect for an important part the genetic structure of *S. cerevisiae* population. Accordingly, the genetic polymorphism may be a contributor to the particular abilities of strains to adapt to their environments and consequently, in the emergence of environment-specific phenotypes, as those observed in this study. Recently, quantitative genetic studies of segregating populations from crosses between strains from divergent lineages, were described as a powerful tool for investigating the genetic determinants of polygenic phenotypes [26], [50]. These approaches may be further developed, using parental strains selected from *S. cerevisiae* population on the basis of our phenotypic database, to identify the genetic architecture of particular physiologic and metabolic traits, including those of technological interest.

## Materials and Methods

### Yeast strains

Seventy-two *S. cerevisiae* strains, all prototrophic except *S. cerevisiae* W303-1A (*MAT*α *leu2-3, 112 ura3-1 trp1-1 his3-11, 15 ade2-1*), collected from ecologically and geographically diverse environments (Table 1) were characterized in this study. Many of the strains came from Washington University (22) and Sanger Institute (36), whereas others were obtained from several different companies or laboratories. The genome sequences of most of these strains are available. According to their origin and/or existing classifications in the Sanger Institute and Washington University databases, our strains were first classified into seven major groups: *baker*, *clinical*, *fermentation processes*, *laboratory*, *vineyard*, *natural* and *wine commercial*. The *fermentation processes* and *natural* clades were further separated into three (*beer*, *palm wine* and *sake*) and five (*oak*, *Bertam palm*, *soil*, *fruit* and *cactus*) sub-groups, respectively. For each strain, an aliquot of a reference stock, conserved at −80°C, was transferred to a YPD agar plate (1% Bacto yeast extract, 2% bactopeptone, 2% glucose, 1.5% agar) 48 h before fermentation.

### Fermentation conditions

Initial cultures in YPD medium were grown in 50 mL flasks at 28°C, with shaking, (150 rpm) for 12 h. These cultures were used to inoculate secondary cultures at a density of 1×10^6^ cells/mL. Fermentations were carried out in synthetic MS medium, which contained 240 g/L glucose, 6 g/L malic acid, 6 g/L citric acid and 200 mg/L nitrogen in the form of amino acids (148 mg N/L) and NH_4_Cl (52 mg N/L), at pH 3.5 (5). Ergosterol (1.875 mg/L), oleic acid (0.625 mg/L) and Tween 80 (0.05 g/L) were provided as anaerobic growth factors. Fermentations took place in 1.1 liter fermentors equipped with fermentation locks to maintain anaerobiosis, at 28°C, with continuous magnetic stirring (500 rpm). The CO_2_ release was followed by automatic measurement of fermentor weight loss every 20 minutes. The rate of CO_2_ production (dCO_2_/dt, where t is time) was calculated by polynomial smoothing of the last ten values of CO_2_ production. The frequent acquisition of CO_2_ release values and highly precise bioreactor weighing (±10 mg) allowed accurate CO_2_ production rates to be calculated, with good repeatability and a small variation coefficient: (dCO_2_/dt)_max_ = 0.8% [31]. For data analysis, five variables were determined from the entire fermentation rate curve. (1) The total amount of CO_2_ released, allowed us to estimate the fermentative capacity of the yeasts and to identify the strains unable to completely ferment the available glucose (240 g/L) (“stuck” profiles). (2 & 3) The times required to ferment 50% (T_50_) and 75% (T_75_) sugars were recorded because some strains displayed “stuck” fermentation profiles and were not able to complete the fermentation process. (4 & 5) Finally, the maximal CO_2_ production rate (V_max_) and the rate at mid-fermentation (V_50_) reflected yeast activity at the beginning of the process and during the stationary phase.

### Analytic methods

Cells were counted using an electronic particle counter (Multisizer 3 Coulter Counter, Beckman Coulter) fitted with a probe with a 100-µm aperture. The dry weight of the yeast was measured by filtering 50 mL of culture though a 0.45 µm-pore Millipore nitrocellulose filter, which was washed twice with 50 mL distilled water and dried for 24 h at 105°C. These analyses were performed at T_75_ (when 75% sugar was consumed).

Glucose and fermentation products (acetate, succinate, glycerol and ethanol) were analyzed by high-pressure liquid chromatography (HPLC 1100, Agilent Technologies) on an HPX-87H Aminex column (Bio-Rad Laboratories Inc.). Dual detection was performed with a refractometer and a UV detector (Hewlett Packard).

The concentrations of volatile compounds were assayed by gas chromatography using an Agilent 6890 chromatograph equipped with an headspace injector and a ZB-WAX column (60 m×0.32 mm×0.5 µm) from Phenomenex Inc. The pressure was held constant (120 kPa) and temperature was progressively increased. The column temperature was: initially held at 38°C for 3 min, then increased to 65°C at a rate of 3°C/min, then increased to 160°C at a rate of 6°C/min, then held at 160°C for 5 min, then increased to 230°C at a rate of 8°C/min, and finally held at 230°C for 5 min. The compounds were detected using a flame ionization detection (FID) system. The final ethanol concentration of the samples was adjusted to 11%, to standardize transfer between the liquid and the headspace.

### Statistical analyses

Statistical analyses were performed using the R software, version 2.9.2.

For each trait, usual descriptive statistics were calculated by strain origin with mean, standard deviation and coefficient of variation.

Reproducibility of measurements was evaluated from data of fermentations achieved at least in duplicate with the same strains (n = 21 strains) using the intra-class correlation coefficient [51].

Pairwise correlations between variables were calculated using Pearson's Product Moment correlation coefficients (r). Since 153 multiple correlations were computed, p-values were corrected for multiple testing using Benjamini-Hochberg methods [52] by means of R's multtest package [53]. As some distributions were not gaussian, the robustness of the Pearson's coefficients was studied comparing this approach with the Spearman's rank correlations. Both approaches were consistent and only Pearson's correlations were presented.

For a global analysis of phenotypic diversity within *S. cerevisiae* population, a principal component analysis [PCA] and a linear discriminant analysis [LDA] (with origin as factor) were first performed considering the whole data set using ade4 package [54].

To analyse the diversity regarding the origin of the strains, the most discriminant variables according to strain origin were selected. Each variables was tested using a one-way ANOVA in order to keep the ones having a significant impact on strain origin (at a p-value threshold of 0.05), without any multiplicity adjustment. For each trait, normality of residual distributions and homogeneity of variance were studied using usual diagnostic graphics. As some traits failed in showing strict homogeneity of variance, a robustness analysis was done using a Kruskall-Wallis test (non-parametric test) to assess a global effect of the strain origin. As the results between the 2 analyses were consistent (Table S5), the one-way ANOVA has been considered as robust and only these results were systematically presented.

To reveal the structure of the population according to the origin of the 72 strains and the chosen phenotypes, a LDA was performed on the selected subset of phenotypic variables using the *discrimin* function of the ade4 package [54].

LDA is a supervised multivariate method that uses the class information to characterize the structure of the data by maximizing the ratio of “between-class” variance to “within-class” variance. The resulting combination was used as a linear classifier for dimensionality reduction, to separate the strains origin according to the studied phenotypes.

## Supporting Information

Figure S1 **Fermentation profile as a phenotypic fingerprint.**
(PDF)

Figure S2 **Analysis of the phenotypic traits of the 72** ***S. cerevisiae*** **strains regarding to their habitat.**
(PDF)

Figure S3 **Overview of the phenotypic diversity between** ***S. cerevisiae*** **strains from diverse habitats.**
(PDF)

Figure S4 **Strain origin-dependent characterization of** ***S. cerevisiae*** **population on the basis of phenotypes.**
(PDF)

Table S1 **Intra-class correlation coefficient (ICC) of the 21 strains which have been grown under wine fermentation conditions at least in duplicate.**
(PDF)

Table S2 **Correlation analysis between the 18 phenotypic traits within the population of 72 stains.**
(PDF)

Table S3 **Correlations between variables within the whole population (72 strains).**
(PDF)

Table S4 **Descriptive statistics of the phenotypic variables regarding the origin of the strains.**
(PDF)

Table S5 **Comparison of the one-way ANOVAs and Kruskal-Wallis tests for the phenotypic variables.**
(PDF)
