# Supplementary material for: Phenotypic Landscape of Saccharomyces cerevisiae during Wine Fermentation: Evidence for Origin-Dependent Metabolic Traits
Source: PLoS One. 2011 Sep 16;6(9):e25147. doi: 10.1371/journal.pone.0025147 (PMC3174997; doi:10.1371/journal.pone.0025147)
Supplement: Figure S1 — Fermentation profile as a phenotypic fingerprint. (PDF) [file pone.0025147.s001.pdf]

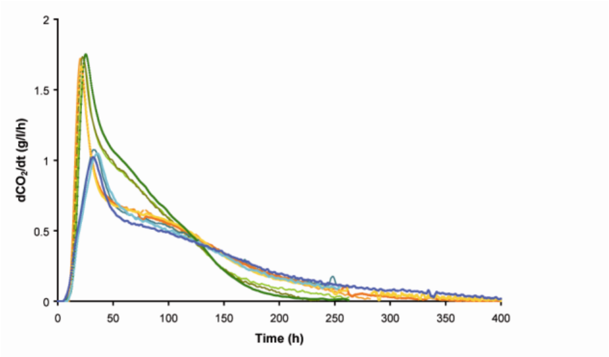

Figure S1. Fermentation profile as a phenotypic fingerprint. Fermentations of the strains T73 (orange), YS9 (green) and Y10 (blue) were carried out in triplicate in synthetic medium containing 240 g/L glucose, 200 mg/L nitrogen, pH 3.5. The CO_2 production rate was on-line monitored by measurement of the loss of fermentor weight and presented as a function of time.
