# Supplementary material for: Phenotypic Landscape of Saccharomyces cerevisiae during Wine Fermentation: Evidence for Origin-Dependent Metabolic Traits
Source: PLoS One. 2011 Sep 16;6(9):e25147. doi: 10.1371/journal.pone.0025147 (PMC3174997; doi:10.1371/journal.pone.0025147)
Supplement: Figure S2 — Analysis of the phenotypic traits of the 72 S. cerevisiae strains regarding to their habitat. (PDF) [file pone.0025147.s002.pdf]

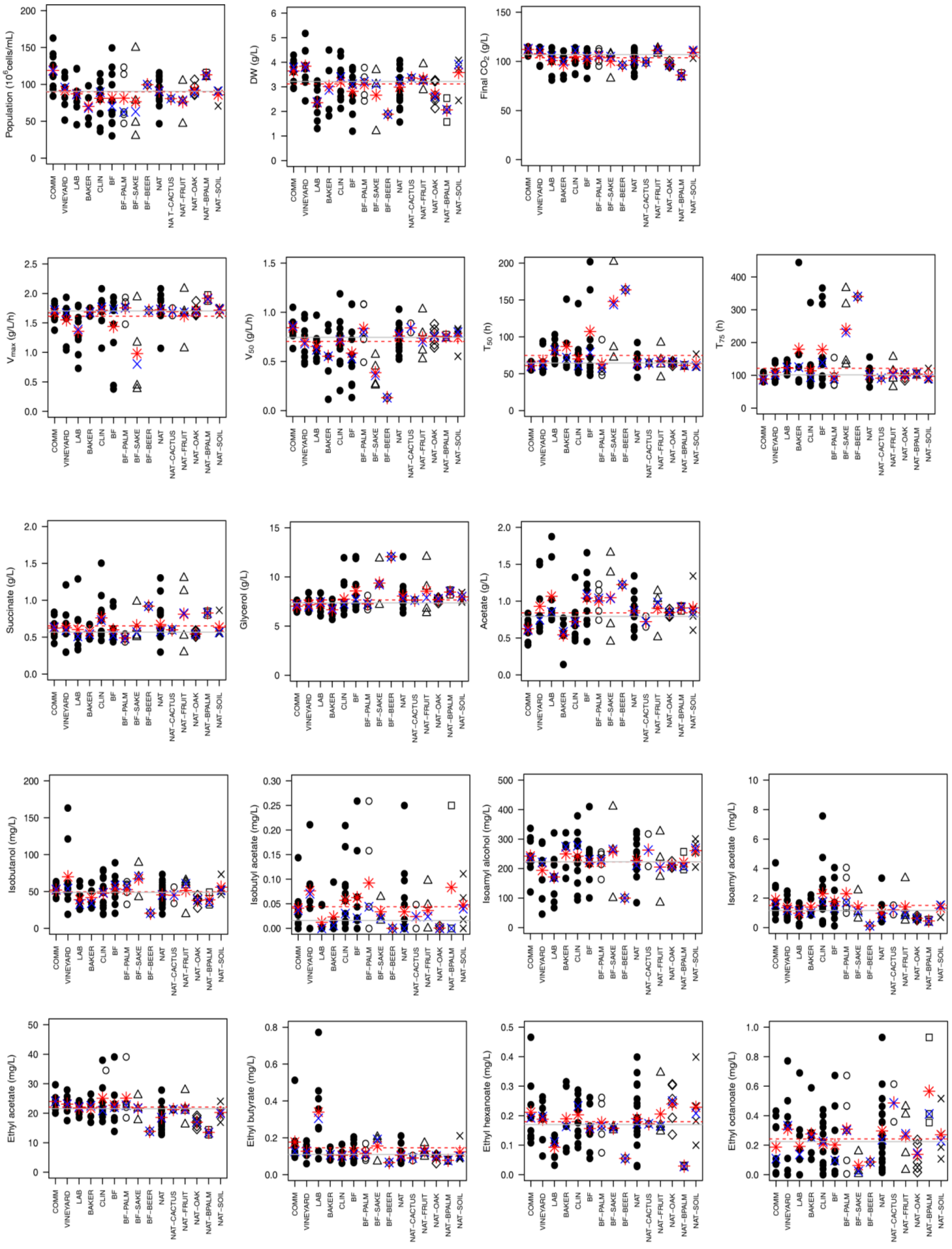

Figure S2 : Analysis of the phenotypic traits of the 72 *S. cerevisiae* strains regarding to their habitat. The 18 measured variables were reported regarding the 7 main groups of origin: *baker*, *clinical*, *fermentation processes*, *laboratory*, *vineyard*, *natural* and *wine commercial* (closed symbols). Open symbols correspond to the subdivision of *fermentation processes* group into *beer*, *palm wine*, *sake* sub-groups and of *natural* groups into *oak*, *Bertam palm*, *soil*, *fruit*, *cactus* sub-groups. The mean and median of the entire population are reported as red and blue lines, respectively. The mean and the median of each groups or sub-groups are indicated by red and blue stars, respectively.
