# Supplementary material for: Phenotypic Landscape of Saccharomyces cerevisiae during Wine Fermentation: Evidence for Origin-Dependent Metabolic Traits
Source: PLoS One. 2011 Sep 16;6(9):e25147. doi: 10.1371/journal.pone.0025147 (PMC3174997; doi:10.1371/journal.pone.0025147)
Supplement: Figure S3 — Overview of the phenotypic diversity between S. cerevisiae strains from diverse habitats. (PDF) [file pone.0025147.s003.pdf]

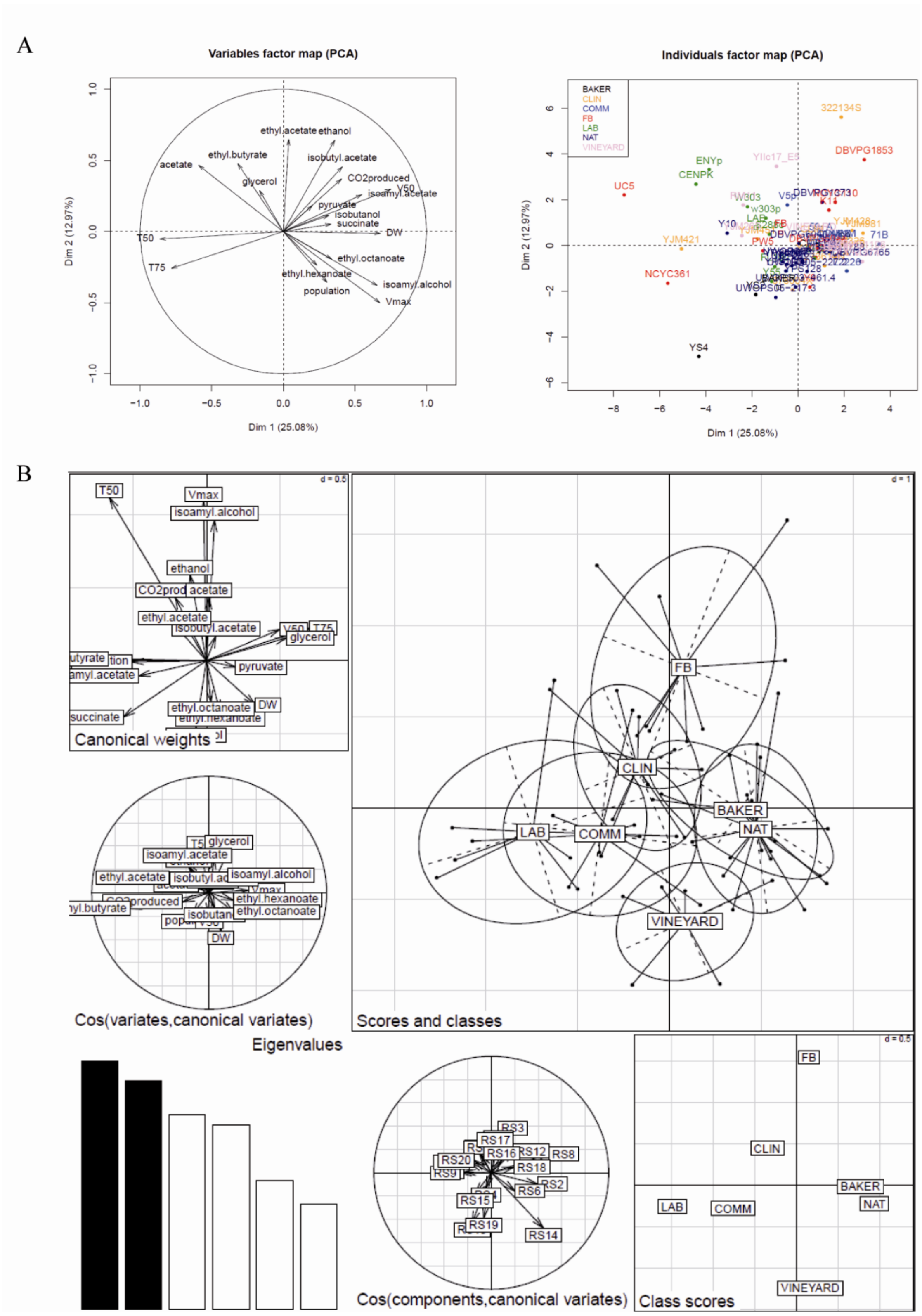

Figure S3. Overview of the phenotypic diversity between *S. cerevisiae* strains from diverse habitats. Principal component analysis PCA (A) and Linear discriminant analysis LDA (B) of all the strains and all the phenotypic variables. (A) X-axis, percentage of variation

explained by principal component 1 (Dim 1) (25.08%); Y-axis, percentage of variation explained by Dim 2 (12.97%). Only 38% of the variation of the dataset is explained by this PCA that does not allowed to separate the strains on the basis of their origin. (B) Plots provided by the *discrimin* function of the ade4 package. The right-upper plot shows the projections of the individuals onto the plane defined by the axes of the LDA. Groups are displayed by ellipses where the centers are the means (between variances) and the ellipses the within variance. Others plots are diagnostic plots of the interpretability of the LDA and show that this LDA is not suitably interpretable. 53.7% of the between-group variation is explained by the two first discriminant axes.
