# Supplementary material for: Phenotypic Landscape of Saccharomyces cerevisiae during Wine Fermentation: Evidence for Origin-Dependent Metabolic Traits
Source: PLoS One. 2011 Sep 16;6(9):e25147. doi: 10.1371/journal.pone.0025147 (PMC3174997; doi:10.1371/journal.pone.0025147)
Supplement: Figure S4 — Strain origin-dependent characterization of S. cerevisiae population on the basis of phenotypes. (PDF) [file pone.0025147.s004.pdf]

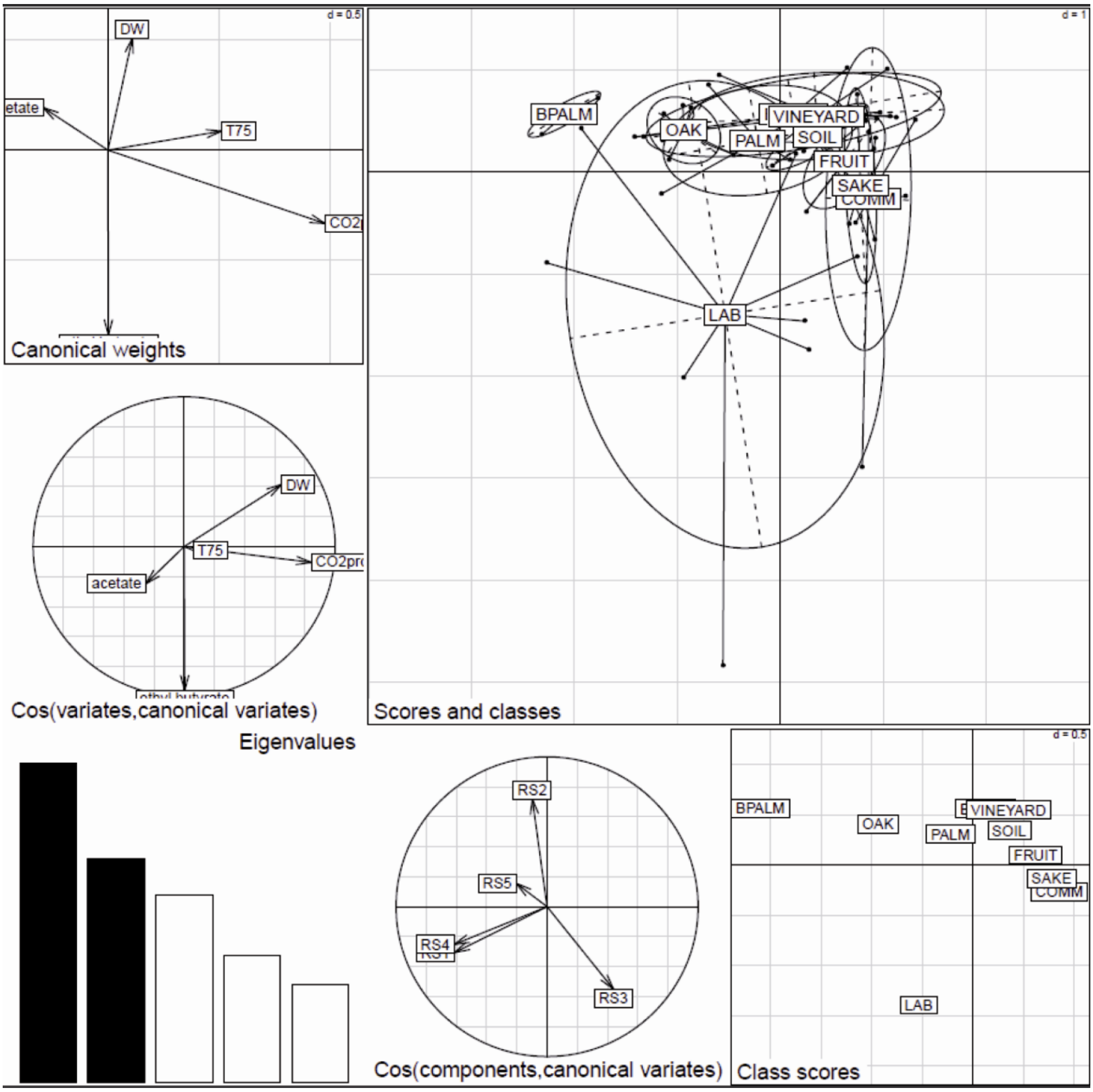

Figure S4: Strain origin-dependent characterization of *S. cerevisiae* population on the basis of phenotypes. Linear discriminant analysis was carried out on the selected phenotypic variables (dry weight, T_{75}, CO_{2F}, acetate and ethyl butyrate) for the description of the strains depending on their origin.
