# Supplementary material for: Phenotypic Landscape of Saccharomyces cerevisiae during Wine Fermentation: Evidence for Origin-Dependent Metabolic Traits
Source: PLoS One. 2011 Sep 16;6(9):e25147. doi: 10.1371/journal.pone.0025147 (PMC3174997; doi:10.1371/journal.pone.0025147)
Supplement: Table S1 — Intra-class correlation coefficient (ICC) of the 21 strains which have been grown under wine fermentation conditions at least in duplicate. (PDF) [file pone.0025147.s005.pdf]

Table SI. Intra-class correlation coefficient (ICC) of the 21 strains which have been grown under wine fermentation conditions at least in duplicate.

Parameter	Between-strain Variance	Within-strain Variance	ICC (%)
Cell number	1219.5	26.6	97.9
Dry weight	0.706	0.033	95.6
V_{max}	0.110	0.006	95.1
CO_{2F}	128.0	11.7	91.6
T_{75}	12024.7	145.0	98.8
Succinate	0.092	0.004	96.1
Glycerol	2.524	0.086	96.7
Acetate	0.126	0.003	97.8
Pyruvate	0.00086	0.000083	91.3
Isobutanol	207.4	14.3	93.6
Isobutyl.acetate	0.0023	0.000033	98.6
Isoamyl.alcohol	6992.5	77.6	98.9
Isoamyl Acetate.	1.093	0.016	98.5
Ethyl acetate	76.4	3.2	96.0
Ethyl butyrate	0.004	0.001	85.7
Ethyl hexanoate	0.0059	0.00021	96.5
Ethyl octanoate	0.035	0.002	94.2
