# Supplementary material for: Phenotypic Landscape of Saccharomyces cerevisiae during Wine Fermentation: Evidence for Origin-Dependent Metabolic Traits
Source: PLoS One. 2011 Sep 16;6(9):e25147. doi: 10.1371/journal.pone.0025147 (PMC3174997; doi:10.1371/journal.pone.0025147)
Supplement: Table S2 — Correlation analysis between the 18 phenotypic traits within the population of 72 stains. (PDF) [file pone.0025147.s006.pdf]

Table S2. Correlation analysis between the 18 phenotypic traits within the population of 72 stains. Pearson's Product Moment correlation coefficients.

	Cell number	**Dry weight**	**V_{max}**	**CO_{2F}**	**T_{75}**	**T_{50}**	**V_{50}**	Succinate	Glycerol	**Acetate**	Isobutanol	**Isobutyl. acetate**	**Isoamyl alcohol**	**Isoamyl acetate**	**Ethyl. acetate**	Ethyl. butyrate	Ethyl. hexanoate	Ethyl. octanoate
Cell number	*1.00*	*0.31*	*0.37*	*0.23*	*-0.32*	*-0.36*	*0.24*	*-0.07*	*-0.23*	*-0.32*	*0.03*	*-0.21*	*0.10*	*-0.24*	*-0.35*	*-0.05*	*0.01*	*0.02*
**Dry weight**	0.31	*1.00*	*0.25*	*0.71*	*-0.47*	*-0.47*	*0.35*	*0.02*	*-0.29*	*-0.34*	*0.35*	*0.12*	*0.37*	*0.19*	*0.01*	*-0.24*	*0.28*	*0.24*
**V_{max}**	0.37	0.25	*1.00*	*0.03*	*-0.52*	*-0.73*	*0.48*	*0.30*	*-0.21*	*-0.56*	*0.03*	*0.14*	*0.50*	*0.33*	*-0.26*	*-0.50*	*0.16*	*0.31*
**CO_{2F}**	0.23	**0.71**	0.03	*1.00*	*-0.46*	*-0.35*	*0.34*	*-0.05*	*-0.19*	*-0.10*	*0.26*	*0.07*	*0.09*	*0.16*	*0.10*	*0.14*	*0.12*	*-0.08*
**T75**	-0.32	-0.47	**-0.52**	-0.46	*1.00*	*0.92*	*-0.81*	*-0.15*	*0.24*	*0.27*	*-0.12*	*-0.23*	*-0.28*	*-0.26*	*0.04*	*-0.03*	*0.03*	*-0.15*
**T50**	-0.36	-0.47	**-0.73**	-0.35	**0.92**	*1.00*	*-0.78*	*-0.21*	*0.34*	*0.42*	*-0.12*	*-0.27*	*-0.37*	*-0.32*	*0.06*	*0.11*	*-0.04*	*-0.23*
**V50**	0.24	0.35	**0.48**	0.34	**-0.81**	**-0.78**	*1.00*	*0.27*	*-0.11*	*-0.30*	*-0.02*	*0.37*	*0.21*	*0.40*	*0.16*	*0.02*	*-0.04*	*0.23*
Succinate	-0.07	0.02	0.30	-0.05	-0.15	-0.21	0.27	*1.00*	*0.55*	*-0.18*	*0.02*	*0.31*	*0.32*	*0.39*	*0.20*	*-0.22*	*-0.09*	*0.08*
Glycerol	-0.23	-0.29	-0.21	-0.19	0.24	0.34	-0.11	0.55	*1.00*	*0.29*	*-0.04*	*0.23*	*-0.05*	*0.12*	*0.18*	*-0.01*	*-0.12*	*-0.12*
**Acetate**	-0.32	-0.34	-0.56	-0.10	0.27	0.42	-0.30	-0.18	0.29	*1.00*	*-0.11*	*-0.03*	*-0.66*	*-0.30*	*0.15*	*0.31*	*-0.27*	*-0.11*
Isobutanol	0.03	0.35	0.03	0.26	-0.12	-0.12	-0.02	0.02	-0.04	-0.11	*1.00*	*0.45*	*0.40*	*0.10*	*0.03*	*-0.13*	*0.07*	*0.06*
Isobutyl.acetate	-0.21	0.12	0.14	0.07	-0.23	-0.27	0.37	0.31	0.23	-0.03	0.45	*1.00*	*0.17*	*0.51*	*0.44*	*-0.14*	*-0.08*	*0.31*
**Isoamyl.alcohol**	0.10	0.37	0.50	0.09	-0.28	-0.37	0.21	0.32	-0.05	**-0.66**	0.40	0.17	*1.00*	*0.49*	*-0.14*	*-0.39*	*0.34*	*0.23*
Isoamyl.acetate	-0.24	0.19	0.33	0.16	-0.26	-0.32	0.40	0.39	0.12	-0.30	0.10	**0.51**	**0.49**	*1.00*	*0.51*	*-0.16*	*0.31*	*0.07*
Ethyl acetate	-0.35	0.01	-0.26	0.10	0.04	0.06	0.16	0.20	0.18	0.15	0.03	**0.44**	-0.14	**0.51**	*1.00*	*0.10*	*0.07*	*0.00*
Ethyl butyrate	-0.05	-0.24	-0.50	0.14	-0.03	0.11	0.02	-0.22	-0.01	0.31	-0.13	-0.14	-0.39	-0.16	0.10	*1.00*	*-0.27*	*-0.21*
Ethyl hexanoate	0.01	0.28	0.16	0.12	0.03	-0.04	-0.04	-0.09	-0.12	-0.27	0.07	-0.08	0.34	0.31	0.07	-0.27	*1.00*	*-0.04*
Ethyl octanoate	0.02	0.24	0.31	-0.08	-0.15	-0.23	0.23	0.08	-0.12	-0.11	0.06	0.31	0.23	0.07	0.00	-0.21	-0.04	*1.00*
