# Supplementary material for: Phenotypic Landscape of Saccharomyces cerevisiae during Wine Fermentation: Evidence for Origin-Dependent Metabolic Traits
Source: PLoS One. 2011 Sep 16;6(9):e25147. doi: 10.1371/journal.pone.0025147 (PMC3174997; doi:10.1371/journal.pone.0025147)
Supplement: Table S3 — Correlations between variables within the whole population (72 strains). (PDF) [file pone.0025147.s007.pdf]

Table S3. Correlations between variables within the whole population (72 strains). Pearson's Product Moment correlation coefficients with p-values corrected for multiple testing using Benjamini-Hochberg methods (BH).

Name	r	rawp	BH
T75-V50	-0.81	3.38E-18	5.18E-16
T75-T50	0.92	2.20E-16	1.68E-14
T50-V50	-0.78	5.20E-16	2.65E-14
Vmax-T50	-0.73	2.31E-13	8.84E-12
DW-CO2produced	0.71	8.38E-12	2.56E-10
acetate-isoamyl.alcohol	-0.66	2.60E-10	6.62E-09
Vmax-acetate	-0.56	2.82E-07	6.17E-06
succinate-glycerol	0.55	4.46E-07	8.54E-06
Vmax-T75	-0.52	2.51E-06	4.26E-05
isobutyl.acetate-isoamyl.acetate	0.51	5.48E-06	8.39E-05
Vmax-isoamyl.alcohol	0.50	7.25E-06	9.24E-05
isoamyl.acetate-ethyl.acetate	0.51	6.81E-06	9.24E-05
Vmax-ethyl.butyrate	-0.50	8.72E-06	0.00010268
isoamyl.alcohol-isoamyl.acetate	0.49	1.71E-05	0.00018673
Vmax-V50	0.48	2.26E-05	0.00023042
DW-T75	-0.47	4.80E-05	0.0004082
DW-T50	-0.47	4.75E-05	0.0004082
CO2produced-T75	-0.46	4.49E-05	0.0004082
isobutanol-isobutyl.acetate	0.45	8.73E-05	0.00070306
isobutyl.acetate-ethyl.acetate	0.44	0.00012165	0.00093065
T50-acetate	0.42	0.00022658	0.00165078
V50-isoamyl.acetate	0.40	0.0004884	0.00339661
isobutanol-isoamyl.alcohol	0.40	0.00059406	0.0039518
succinate-isoamyl.acetate	0.39	0.0006962	0.00426077
isoamyl.alcohol-ethyl.butyrate	-0.39	0.00067902	0.00426077
V50-isobutyl.acetate	0.37	0.0014847	0.00873687
T50-isoamyl.alcohol	-0.37	0.0016531	0.00936754
DW-isoamyl.alcohol	0.37	0.00173962	0.00950581
population-Vmax	0.37	0.00219919	0.01160262
CO2produced-T50	-0.35	0.00242471	0.01236601
DW-V50	0.35	0.00258486	0.01275754
DW-isobutanol	0.35	0.00318203	0.0152141
population-T50	-0.36	0.00346207	0.01582771
T50-glycerol	0.34	0.00351727	0.01582771
DW-acetate	-0.34	0.00368032	0.01608825
CO2produced-V50	0.34	0.00381292	0.0162049
population-ethyl.acetate	-0.35	0.00409832	0.01694709
isoamyl.alcohol-ethyl.hexanoate	0.34	0.00421404	0.01696705
Vmax-isoamyl.acetate	0.33	0.00444669	0.01744472
T50-isoamyl.acetate	-0.32	0.00609248	0.02330373

Vmax-ethyl.octanoate	0.31	0.00756637	0.0269524
succinate-isoamyl.alcohol	0.32	0.00726073	0.0269524
acetate-ethyl.butyrate	0.31	0.00757486	0.0269524
isoamyl.acetate-ethyl.hexanoate	0.31	0.00791331	0.02751674
population-T75	-0.32	0.00938806	0.02953627
population-acetate	-0.32	0.00879644	0.02953627
Vmax-succinate	0.30	0.00962104	0.02953627
V50-acetate	-0.30	0.00965238	0.02953627
succinate-isobutyl.acetate	0.31	0.00943373	0.02953627
isobutyl.acetate-ethyl.octanoate	0.31	0.00888196	0.02953627
acetate-isoamyl.acetate	-0.30	0.01041965	0.03125894
population-DW	0.31	0.01148029	0.03377854
glycerol-acetate	0.29	0.01329906	0.03839161
DW-glycerol	-0.29	0.01567506	0.04441268
T75-isoamyl.alcohol	-0.28	0.01741277	0.04843916
DW-ethyl.hexanoate	0.28	0.0178101	0.04865973
T75-acetate	0.27	0.01953355	0.05243216
V50-succinate	0.27	0.0205365	0.05417386
ethyl.butyrate-ethyl.hexanoate	-0.27	0.02130352	0.05524472
T50-isobutyl.acetate	-0.27	0.02450537	0.0615924
acetate-ethyl.hexanoate	-0.27	0.02455645	0.0615924
Vmax-ethyl.acetate	-0.26	0.03012444	0.07433935
CO2produced-isobutanol	0.26	0.03106173	0.07543563
T75-isoamyl.acetate	-0.26	0.03166574	0.0757009
DW-Vmax	0.25	0.03379292	0.07954334
DW-ethyl.octanoate	0.24	0.04108357	0.09523919
DW-ethyl.butyrate	-0.24	0.04568613	0.1027938
T75-glycerol	0.24	0.04522077	0.1027938
T75-isobutyl.acetate	-0.23	0.05123216	0.11304992
isoamyl.alcohol-ethyl.octanoate	0.23	0.05172218	0.11304992
population-isoamyl.acetate	-0.24	0.05446135	0.11573036
T50-ethyl.octanoate	-0.23	0.05400456	0.11573036
V50-ethyl.octanoate	0.23	0.05558368	0.11649729
population-V50	0.24	0.05926318	0.12089689
glycerol-isobutyl.acetate	0.23	0.05916566	0.12089689
succinate-ethyl.butyrate	-0.22	0.06077358	0.12234681
population-glycerol	-0.23	0.06223834	0.12366838
population-CO2produced	0.23	0.0659703	0.12940328
Vmax-glycerol	-0.21	0.07228952	0.14000376
T50-succinate	-0.21	0.08252405	0.15591353
V50-isoamyl.alcohol	0.21	0.08254246	0.15591353
ethyl.butyrate-ethyl.octanoate	-0.21	0.08559831	0.15971392
population-isobutyl.acetate	-0.21	0.09639699	0.17769565
succinate-ethyl.acetate	0.20	0.10175253	0.18533497

CO2produced-glycerol	-0.19	0.10852111	0.19533799
DW-isoamyl.acetate	0.19	0.11131124	0.19803047
succinate-acetate	-0.18	0.12317648	0.2166207
glycerol-ethyl.acetate	0.18	0.14074051	0.24469657
isobutyl.acetate-isoamyl.alcohol	0.17	0.147897	0.25424989
isoamyl.acetate-ethyl.butyrate	-0.16	0.17042245	0.28971816
CO2produced-isoamyl.acetate	0.16	0.19073275	0.32068254
Vmax-ethyl.hexanoate	0.16	0.19507336	0.32092714
V50-ethyl.acetate	0.16	0.1936902	0.32092714
T75-ethyl.octanoate	-0.15	0.19996653	0.32547743
T75-succinate	-0.15	0.21581662	0.34757835
acetate-ethyl.acetate	0.15	0.2237751	0.35664156
isobutyl.acetate-ethyl.butyrate	-0.14	0.23725206	0.37422232
isoamyl.alcohol-ethyl.acetate	-0.14	0.24169676	0.3773429
CO2produced-ethyl.butyrate	0.14	0.24514717	0.3788638
Vmax-isobutyl.acetate	0.14	0.25507418	0.39026349
isobutanol-ethyl.butyrate	-0.13	0.29132845	0.44131933
T50-isobutanol	-0.12	0.3025663	0.45384945
DW-isobutyl.acetate	0.12	0.31732861	0.4668392
glycerol-ethyl.octanoate	-0.12	0.31681015	0.4668392
glycerol-ethyl.hexanoate	-0.12	0.32171448	0.46878395
T75-isobutanol	-0.12	0.33034625	0.47682054
CO2produced-ethyl.hexanoate	0.12	0.33828681	0.47923965
glycerol-isoamyl.acetate	0.12	0.33737968	0.47923965
T50-ethyl.butyrate	0.11	0.34578264	0.48536462
acetate-isobutanol	-0.11	0.35849713	0.49863691
acetate-ethyl.octanoate	-0.11	0.36748686	0.50653594
V50-glycerol	-0.11	0.38002972	0.51914774
ethyl.acetate-ethyl.butyrate	0.10	0.38901179	0.52671507
CO2produced-ethyl.acetate	0.10	0.4072248	0.54653855
CO2produced-acetate	-0.10	0.41347836	0.55010599
population-isoamyl.alcohol	0.10	0.42479272	0.55549817
isobutanol-isoamyl.acetate	0.10	0.42221167	0.55549817
CO2produced-isoamyl.alcohol	0.09	0.45507908	0.59006017
succinate-ethyl.hexanoate	-0.09	0.46387949	0.59641648
CO2produced-ethyl.octanoate	-0.08	0.49788593	0.62819382
succinate-ethyl.octanoate	0.08	0.50091272	0.62819382
isobutyl.acetate-ethyl.hexanoate	-0.08	0.50070306	0.62819382
CO2produced-isobutyl.acetate	0.07	0.537593	0.66871324
ethyl.acetate-ethyl.hexanoate	0.07	0.54969501	0.67825271
isoamyl.acetate-ethyl.octanoate	0.07	0.57866819	0.70828986
isobutanol-ethyl.hexanoate	0.07	0.58951677	0.71584179
population-succinate	-0.07	0.60541354	0.72935647
isobutanol-ethyl.octanoate	0.06	0.6103416	0.72954894

T50-ethyl.acetate	0.06	0.61750631	0.7323912
CO2produced-succinate	-0.05	0.65953374	0.77622048
population-ethyl.butyrate	-0.05	0.66826552	0.78049332
glycerol-isoamyl.alcohol	-0.05	0.69454248	0.80503787
Vmax-isobutanol	0.03	0.77729963	0.85558881
T75-ethyl.acetate	0.04	0.77095708	0.85558881
T50-ethyl.hexanoate	-0.04	0.76676638	0.85558881
V50-ethyl.hexanoate	-0.04	0.74528263	0.85558881
glycerol-isobutanol	-0.04	0.76597702	0.85558881
isobutanol-ethyl.acetate	0.03	0.77319092	0.85558881
ethyl.hexanoate-ethyl.octanoate	-0.04	0.76056354	0.85558881
T75-ethyl.butyrate	-0.03	0.78936599	0.86266426
population-isobutanol	0.03	0.80433033	0.87278398
T75-ethyl.hexanoate	0.03	0.81629404	0.87952808
Vmax-CO2produced	0.03	0.83214215	0.88415104
acetate-isobutyl.acetate	-0.03	0.82902882	0.88415104
V50-isobutanol	-0.02	0.83989149	0.88623033
DW-succinate	0.02	0.85131283	0.8921292
population-ethyl.octanoate	0.02	0.87610387	0.90239846
V50-ethyl.butyrate	0.02	0.87115774	0.90239846
succinate-isobutanol	0.02	0.87880634	0.90239846
population-ethyl.hexanoate	0.01	0.91437689	0.93266443
glycerol-ethyl.butyrate	-0.01	0.94208523	0.95456318
DW-ethyl.acetate	0.01	0.95594346	0.96223256
ethyl.acetate-ethyl.octanoate	0.00	0.97025101	0.97025101
