# Supplementary material for: Phenotypic Landscape of Saccharomyces cerevisiae during Wine Fermentation: Evidence for Origin-Dependent Metabolic Traits
Source: PLoS One. 2011 Sep 16;6(9):e25147. doi: 10.1371/journal.pone.0025147 (PMC3174997; doi:10.1371/journal.pone.0025147)
Supplement: Table S4 — Descriptive statistics of the phenotypic variables regarding the origin of the strains. (PDF) [file pone.0025147.s008.pdf]

Table S4. Descriptive statistics of the phenotypic variables regarding the origin of the strains.

Origin	Baker	Clinical	Wine commercial	Fermentation processes	Laboratory	Natural	Vineyard	Total
Cell number	69.98 ±24.2 (35%)	81.08 ±26.98 (33%)	119.11 ±27.3 (23%)	81.13 ±38.83 (48%)	87.1 ±25.36 (29%)	90.95 ±17.02 (19%)	91.67 ±21.92 (24%)	89.71 ±27.86 (31%)
Dry weight	3.01 ±1.28 (43%)	3.2 ±0.71 (22%)	3.64 ±0.5 (14%)	2.82 ±0.86 (31%)	2.35 ±0.7 (30%)	2.99 ±0.71 (24%)	3.84 ±0.86 (22%)	3.12 ±0.84 (27%)
Vmax	1.68 ±0.05 (3%)	1.72 ±0.24 (14%)	1.67 ±0.15 (9%)	1.44 ±0.59 (41%)	1.36 ±0.38 (28%)	1.72 ±0.2 (12%)	1.55 ±0.32 (20%)	1.61 ±0.33 (21%)
CO_{2F}	96.66 ±12.34 (13%)	104.75 ±9.68 (9%)	112.22 ±2.81 (2%)	102.11 ±8.98 (9%)	100.87 ±11.89 (12%)	101.01 ±9.38 (9%)	107.9 ±7.87 (7%)	103.69 ±9.8 (9%)
T_{75}	179.56 ±151.55 (84%)	113.65 ±64.95 (57%)	88.48 ±10.32 (12%)	178.22 ±116.47 (65%)	123.5 ±16.73 (14%)	99.62 ±18.52 (19%)	109.04 ±27.11 (25%)	120.93 ±70.08 (58%)
T_{50}	86.92 ±38.1 (44%)	70.06 ±24.62 (35%)	61.02 ±4.6 (8%)	107.11 ±67.04 (63%)	81.73 ±14.26 (17%)	64.59 ±8.98 (14%)	66.84 ±16.45 (25%)	74.74 ±32.39 (43%)
V_{50}	0.55 ±0.31 (56%)	0.71 ±0.24 (33%)	0.84 ±0.11 (14%)	0.58 ±0.33 (57%)	0.65 ±0.15 (24%)	0.76 ±0.12 (16%)	0.7 ±0.2 (28%)	0.7 ±0.22 (31%)
Ethanol	81.96 ±2.98 (4%)	85.24 ±1.91 (2%)	85.25 ±1.05 (1%)	86.07 ±2.22 (3%)	84.79 ±1.91 (2%)	84.4 ±1.97 (2%)	85.03 ±1.69 (2%)	84.83 ±2.1 (2%)
Succinate	0.57 ±0.09 (16%)	0.76 ±0.29 (38%)	0.62 ±0.14 (22%)	0.6 ±0.19 (32%)	0.6 ±0.32 (53%)	0.67 ±0.24 (36%)	0.63 ±0.29 (47%)	0.65 ±0.24 (37%)
Glycerol	6.66 ±0.8 (12%)	7.74 ±1.58 (20%)	7.02 ±0.44 (6%)	8.58 ±1.99 (23%)	7.25 ±0.73 (10%)	8.05 ±1.16 (14%)	7.16 ±0.68 (10%)	7.65 ±1.32 (17%)
Acetate	0.54 ±0.24 (45%)	0.72 ±0.22 (31%)	0.62 ±0.14 (23%)	1.04 ±0.36 (35%)	1.06 ±0.43 (40%)	0.87 ±0.18 (21%)	0.93 ±0.39 (42%)	0.84 ±0.31 (37%)
Pyruvate	0.11 ±0.02 (22%)	0.1 ±0.03 (26%)	0.12 ±0.02 (16%)	0.13 ±0.05 (40%)	0.12 ±0.04 (33%)	0.12 ±0.02 (21%)	0.12 ±0.03 (28%)	0.12 ±0.03 (28%)
Isobutanol	41.64 ±14.16 (34%)	47.78 ±21.44 (45%)	51.96 ±7.82 (15%)	53.93 ±20.51 (38%)	38.97 ±14.97 (38%)	45.46 ±13.79 (30%)	70.01 ±47.64 (68%)	49.55 ±22.74 (46%)
Isobutyl acetate	0.02 ±0.04 (173%)	0.06 ±0.07 (116%)	0.04 ±0.04 (104%)	0.06 ±0.09 (143%)	0.01 ±0.02 (178%)	0.03 ±0.06 (187%)	0.08 ±0.06 (81%)	0.04 ±0.06 (139%)
Isoamyl alcohol	249.38 ±62.81 (25%)	241.79 ±85.46 (35%)	242.29 ±63.56 (26%)	220.81 ±95.06 (43%)	171.3 ±83.58 (49%)	227.39 ±55.5 (24%)	193.77 ±86.71 (45%)	222.52 ±76.07 (34%)
Isoamyl acetate	1.38 ±0.64 (46%)	2.29 ±2.02 (88%)	1.9 ±1.21 (64%)	1.76 ±1.32 (75%)	0.98 ±0.56 (57%)	0.97 ±0.7 (73%)	1.35 ±0.74 (55%)	1.5 ±1.25 (83%)
Ethyl acetate	21.77 ±3.84 (18%)	25.08 ±10.27 (41%)	23.99 ±2.66 (11%)	22.74 ±7.1 (31%)	21.81 ±1.97 (9%)	18.55 ±3.85 (21%)	23.1 ±3.13 (14%)	22.07 ±6.03 (27%)
Ethyl butyrate	0.11 ±0.03 (26%)	0.11 ±0.02 (22%)	0.18 ±0.13 (73%)	0.12 ±0.05 (43%)	0.34 ±0.22 (64%)	0.11 ±0.03 (33%)	0.13 ±0.04 (29%)	0.15 ±0.11 (78%)
Ethyl hexanoate	0.19 ±0.11 (60%)	0.2 ±0.07 (32%)	0.21 ±0.11 (53%)	0.16 ±0.07 (43%)	0.09 ±0.04 (40%)	0.19 ±0.1 (55%)	0.19 ±0.07 (36%)	0.18 ±0.09 (50%)
Ethyl octanoate	0.28 ±0.19 (69%)	0.21 ±0.16 (76%)	0.19 ±0.18 (95%)	0.2 ±0.23 (116%)	0.19 ±0.23 (122%)	0.3 ±0.23 (78%)	0.31 ±0.26 (84%)	0.24 ±0.21 (87%)

Mean ± standard deviation (CV%)
