# Supplementary material for: Phenotypic Landscape of Saccharomyces cerevisiae during Wine Fermentation: Evidence for Origin-Dependent Metabolic Traits
Source: PLoS One. 2011 Sep 16;6(9):e25147. doi: 10.1371/journal.pone.0025147 (PMC3174997; doi:10.1371/journal.pone.0025147)
Supplement: Table S5 — Comparison of the one-way ANOVAs and Kruskal-Wallis tests for the phenotypic variables. (PDF) [file pone.0025147.s009.pdf]

Table S5. Comparison of the one-way ANOVAs and Kruskal-Wallis tests for the phenotypic variables. The 72 strains were classified into 7 and 13 groups of origins. The p-values for each phenotypic parameter were reported in the table.

Phenotypic variable	7 groups		13 groups	
	ANOVA	Kruskal-Wallis	ANOVA	Kruskal-Wallis
Cell number	0.035	0.10	0.15	0.17
Dry weight	**0.004**	**0.012**	**0.002**	**0.011**
V_{max}	0.06	0.24	0.004	0.15
V_{50}	0.09	0.10	0.005	0.06
T_{75}	**0.02**	**0.045**	**0.0006**	**0.028**
T_{50}	0.01	0.055	**$4 .10^{-5}$**	**0.025**
CO_{2F}	**0.02**	**0.006**	**0.0005**	**0.0015**
Glycerol	**0.03**	**0.012**	**0.001**	**0.023**
Acetate	**0.001**	**0.0006**	**0.04**	**0.0085**
Succinate	0.65	0.39	0.46	0.19
Isobutanol	0.12	0.39	0.22	0.22
Isobutyl acetate	0.34	0.094	0.33	0.14
Isoamyl alcohol	0.34	0.41	0.46	0.53
Isoamyl acetate	0.06	0.086	0.16	0.038
Ethyl acetate	0.08	0.0077	0.08	0.0064
Ethyl butyrate	**$3. 10^{-6}$**	**0.0037**	**0.0004**	**0.007**
Ethyl hexanoate	0.09	0.057	**0.007**	**0.009**
Ethyl octanoate	0.71	0.59	0.14	0.25

No adjustement of multiplicity.
Variables significant at a 5% level for 7 groups and 13 groups of origins, with the 2 approaches were kept.
